# Patients’ satisfaction with clinical Laboratory Services in Public Hospitals in Ethiopia

**DOI:** 10.1186/s12913-019-4880-9

**Published:** 2020-01-03

**Authors:** Hiwot Amare Hailu, Adinew Desale, Anteneh Yalew, Habtamu Asrat, Sisay Kebede, Daniel Dejene, Hiwot Abebe, Andargachew Gashu, Dereje Yenealem, Birhan Moges, Nebiyou Yemanebrhane, Daniel Melese, Ashebir Gurmessa, Awad Mohammed, Zekaryas Getu, Gonfa Ayana, Adisu Kebede, Ebba Abate

**Affiliations:** 1 Ethiopian Public Health Institute, P.O.BOX: 1242/5654, Addis Ababa, Ethiopia; 2 ILEX Biotech Ltd, CRO Ethiopia, Addis Ababa, Ethiopia; 3 Department of Statistics, College of Natural and Computational Sciences, Addis Ababa University, Addis Ababa, Ethiopia; 4 Division of Epidemiology and Biostatistics, Department of Global Health, Faculty of Medicine and Health Sciences, Stellenbosch University, Cape Town, South Africa

**Keywords:** Patient satisfaction, Hospital, Laboratory service, National survey

## Abstract

**Background:**

Knowing customers’ level of satisfaction is relevant to improve and provide quality health care services. In the clinical laboratory, monitoring customers’ satisfaction is an important indicator of the quality management system and required by international laboratory standards. However, in Ethiopia, there has not been baseline data about the satisfaction level of patients’ with laboratory services at the national level. The aim of this national level survey was to assess patients’ satisfaction level with laboratory services at public hospitals in Ethiopia.

**Methods:**

A national survey was conducted using an institutional based cross-sectional study design was employed from 01 to 30 November 2017. A total of 2399 patients were selected randomly from 60 public hospitals. Data was collected using structured questionnaire, entered in Epi Info and analyzed with SPSS software. Multiple logistic regression model was fitted to identify predictors of patients’ satisfaction with laboratory services. A *p*-value of less than 0.05 was taken as statistically significant.

**Result:**

Overall, 78.6% of the patients were satisfied with the clinical laboratory services. Patients were dissatisfied with cleanness of latrine (47%), long waiting time (30%), clear and understandable advisory service during specimen collection (26%), adequacy of waiting area (25%), easy accessibility of laboratory (19%) and latrine location (20%), availability of requested service (18%), unfair payment of service (17%) and missing of result (12%). The educational status (*P* = 0.032), and distance (*P* = 0.000) were significantly associated with client overall satisfaction level.

**Conclusion:**

Most laboratory patients’ were satisfied with the service provided by public hospital laboratories in public hospitals in Ethiopia. However, patients’ were dissatisfied with the accessibility of sites, adequacy of waiting area, cleanness of latrine, long TAT, communication, missing of results, availability of requested service and cost of service. Therefore, responsible bodies in each level should act on the identified gaps and improve the need of patients in each hospital laboratory. In addition, all hospital laboratories should conduct a satisfaction survey and meet the needs of laboratory patients.

## Background

Medical laboratory service is a critical component of the quality health care system and provides essential data for diagnosing diseases, guiding treatment, determining drug resistance, disease prevention and control, identifying diseases of public health significance through surveillance and public health policy development. An integrated, tiered, functional and sustainable laboratory service is necessary to address these health system needs [1–3].

Satisfaction is the client’s perception of care received compared with the care expected [4]. Evaluating to what extent patients are satisfied with health services is clinically relevant, as satisfied patients are more likely to comply with treatment [5], take an active role in their own care [6], continue using medical care services and implement recommendation of health care providers and maintain with a specific system [5]. On the other hand, clients who are not satisfied with a service may have worse outcomes than others because they miss more appointments, live against the advice or fail to follow through on treatment plans [7]..

The World Health Organization (WHO) indicates that evaluations of client satisfaction might address various aspects of the provided services: reliability and consistency of the services, the responsiveness of services, and the willingness of providers to meet client’s expectations and needs. Thus, the efficiency of services given at laboratories could be measured from a different perspective [8].

In clinical laboratory, monitoring patients’ satisfaction is an important indicator of the quality management system and required by laboratory quality standards, such as ISO 15189: 2012 and ISO17025: 2017. A study shows that satisfied clients are more likely to comply with prescribed treatment and advice, return for additional care when necessary and more willing to pay for services [9]. Studies conducted in developing countries including Ethiopia by the World Bank showed that the level of client satisfaction is low [10].

Comprehensive quality laboratory services are challenging processes that need multiple sources of support from patients, clinical service providers, managers, laboratory professionals, and other stakeholders. Especially, the needs and preference of clients in the clinical laboratory must be addressed in the design and implementation of laboratory quality system. Laboratory clients’ are the best source of information on both the quality and quantity of health care services provided and their views are determining factors in planning and evaluating satisfaction [11].

A number of factors have been shown to influence patients’ satisfaction with health care services including patients’ socio-demographic characters, physical health status, patients’ personal understanding and expectations from various health care services [12]. The general physical appearance of the hospital, as well as the general environment of the premises, also influences the overall satisfaction of the client [13]. The problems are aggravated particularly at peripheral level due to lack of properly designed laboratory rooms, shortage of short term and long term training for laboratory staff, lack of water and electricity, shortage of equipment and supplies, the absence of effective maintenance and spare parts and lack of follow-up and supervision [14].

Therefore, clinical laboratories are expected to assess their patients’ satisfaction level with the laboratory services and is required by laboratory quality standards to improve the service. However, there was no information or data at the national level regarding laboratory patients’ satisfaction level.

### Study objective

The aim of this study was to assess the patients’ satisfaction level with laboratory services at public hospitals in Ethiopia.

## Methods

### Study design and area

An institutional based cross-sectional study design was carried out from November 1 to 30, 2017. Based on the 2015 prediction report, Ethiopia has a total population of 90,074,000. According to Minister of Health 2015 report, there were 234 government hospitals with functional laboratory service, 3547 public health centers and 16,447 health posts in Ethiopia [15]. These health facilities provide different clinical and laboratory services to the community. Public hospital laboratories provid different services that include ART monitoring, microbiology, parasitology, serology, electrolyte, hormone analysis, and other tests for more than 190,000 patients per month in average.

### Source population

All the patients who received laboratory services from public hospitals were source populations.

### Study population and inclusion criteria

All patients who received laboratory services during the study period at selected public hospitals were the study populations. Patients who were less than 15 years old and seriously ill, their adult caregivers who accompanied them were recruited as respondents and included in the study. Critically ill patients who could not able to provide a response and did not have any caretaker at the time of the study were excluded.

### Sample size and sampling procedure

Sample size determination was used for hospital-based assessment surveys by stratified random sampling design (region and hospital types). It is given by

$$ n=\left[\frac{z_{\raisebox{1ex}{$\alpha $}\!\left/ \!\raisebox{-1ex}{$2$}\right.}^2 pq+M{E}^2}{M{E}^2+\frac{z_{\raisebox{1ex}{$\alpha $}\!\left/ \!\raisebox{-1ex}{$2$}\right.}^2 pq}{N}}\Big)\right]\ast d $$

Where n is the sample size to be determined, the value of Z for 5% level of significance is 1.96, *p* = 50% and d is design effect (1.6), ME is margin of allowable error (0.15) [16].

The required sample size of patient was determined by the following formula:

$$ \mathrm{n}={\mathrm{deft}}^2\frac{\left(1/\mathrm{p}-1\right)}{\upalpha^2} $$

Where, p is the assumed value of the population proportion of the underlying variable defining the main indicator of the survey coverage. The proportion of patients satisfaction with laboratory services was 60.4%, according to a study done at Nekemt Referral Hospital [17], deft^2^ = deff = 2 is the design effect, α is the specified relative standard error equals to 0.025 patients, at 95% confidence level and it’s a good relative precision of the indicator at domain estimate level [18]), and response rate is the expected response rate of the survey was 90% for customer survey and as individual response rate.

The sample size in this study was 2399 patients from 60 hospitals. Allocation of the total sample sizes to the regions and hospital types were considered. Since some regions and hospital types are few in size, we applied a power allocation to get guarantee a sufficient sample size in small regions and hospital types in size.

If the desired precision is required at domain/stratum/classification of patients’ satisfaction level, by assuming equal relative variations across strata, a power allocation with an appropriate power value α (0 ≤ α ≤ 1) may be used to guarantee sufficient sample size in small domains/strata [19]. A power value of 1 gives proportional allocation; a power value of 0 gives equal size allocation; a power value between 0 and 1 gives an allocation between proportional allocation and equal size allocation. In our case, we considered α = 0.8. That is ${n}_h=\frac{n\ast {N}_h^{\alpha }}{\sum_{h=1}^J{N}_h^{\alpha }},$ where n =2399, *N*_*h*_= total number of patients in each type of hospitals. Stratified random sampling was used for selection of public hospitals within region and hospital type whereas systematic random sampling was applied for selection of patients for the selected hospitals.

#### Variables

The dependent variable was the level of patients’ satisfaction with clinical laboratory services whereas the independent variables were the factors that affects the satisfaction patients (waiting time, easily accessibility of the laboratory, latrine, courtesy of the laboratory staff, critical value notification, courier service, the reliability of test results, provision of timely test results and others variables).

### Data collection procedures

Data was collected using a pre-tested, structured and interviewer-administered questionnaire. The questionnaire includes variables on socio-demographic and economic data, the length of time to take results, the availability of laboratory staff on working hours, location and cleanness of health institution (latrine and waiting areas) and others.

The satisfaction level was measured using a 5-point Likert scales ranging from very dissatisfied to very satisfied (1 to 5 points). The questionnaire was prepared in English and then translated into local languages. All local language versions of the questionnaire were used for data collection. The questionnaire was pre-tested in similar settings, not included in the study. Data collectors and supervisors were recruited and then trained on the objective of the study and methods of the survey.

### Operational definition

#### Satisfaction level

Client’s perception of the degree to which the customer’s requirements have been fulfilled. It can vary from high satisfaction to low satisfaction. If customers believe that you have met their requirements, they experience high satisfaction. If they believe that you have not met their requirements, they experience low satisfaction.

### Data quality assurance

Data collectors and supervisors were trained on how to select study participants and collect data. After the training of the data collectors, the questionnaire was pretested to ensure the acceptability, comprehensibility, and understandability of the questions by the interviewers. Regular supervision, spot checking and reviewing the completed questionnaire was carried out daily by regional supervisors. Double entry of 15% of the data was carried out.

### Data entry and analysis

Data were entered using Epi Info version 7.2 and analyzed using SPSS version 23. Descriptive statistics were computed to describe data. A 5-point Likert Scale rating of very dissatisfied (1-point), dissatisfied (2-points), neutral (3-points), satisfied (4-points) and very satisfied (5-point) was used. The mean score of satisfaction for each participant was calculated as the average of all satisfaction items.A mean score of 3 and less than 3 was taken as an indicator of participants’ perceived dissatisfaction and a score of more than 3 was taken as the participant was satisfied.

Binary logistic regression model was fitted to identify predictors of customers’ satisfaction with laboratory services. Those variables significant at a *P* value of 0.20 in the crude analysis were included in multiple regression model. A *p*-value of less than 0.05 was used to determine statistically significant. Adjusted Odds Ratio (AOR) with 95% confidence interval (CI) was calculated to identify factors affecting satisfaction level of laboratory customers.

### Ethical consideration

Ethical clearance was obtained from the Scientific and Ethical Review Committee of the Ethiopian Public Health Institution (EPHI). An official permission letter was delivered to the respective regional health bureaus by EPHI during the field work. The facility administration was informed about the general objective and significance of the study through an official letter. Data were collected anonymously, without any personal identifiers. For the purpose of data collection, the aim of the study was explained, and informed consent was obtained from study participants before administering the questions. All participants were informed of their right to refuse the interview at any time.

## Results

### Characteristics of participated health facilities

Sixty public hospitals (31 primary, 20 general, 6 Referral and 3 specialized hospitals) were selected and included in this national survey. Two thousand three hundred ninety-nine patients were selected from the public hospitals and all of them were participated in this survey. These patients were selected from primary hospitals (756), general hospitals (913), referral hospitals (464), and specialized hospitals (266). As the level of hospital increased the number of tests provided to customers was also increased. Out of surveyed health facilities, 78.3% (49) of them could not provide uninterrupted service in the previous one year due to reagent stock out (87.8% (43)) and machine downtime (12.2% (6)).

### Socio-demographic characteristics of the participants

Among the study participants, 53.1% of them were females and 73.2% were married. The mean age and standard deviation of the study participants were 34.19, 13.05 years, respectively. Approximately, 60% of the participants were urban residence. Regarding educational status, 25.8% of them were illiterate, half of them were primary and secondary school completers and 23.5% had college and above educational level. Almost one-third of the respondents were farmers, 19% were government employed, 21.5% were engaged in private sectors and 29.6% were with other occupations and unemployed. Nearly, half of the participants came to the laboratory for the first time to get the service. Detail summary result is described in Table 1.

**Table 1:** Distribution of socio-demographic characteristics of respondents at selected hospitals in Ethiopia, November 2017

Characteristics	Number (*n* = 2399)	Percent
Sex		
Male	1124	46.9
Female	1275	53.1
Marital Status		
Single	642	26.8
Married	1757	73.2
Residence		
Urban	1425	59.4
Rural	974	40.6
Educational Status		
Illiterate	619	25.8
Primary School	675	28.1
Secondary School	542	22.6
College and Above	563	23.5
Occupation		
Farmer	716	29.8
Gov’t Employed	457	19.0
Private Employed	516	21.5
Others	710	29.6
Number of Visits		
First visit	1209	50.4
Two visits	659	27.5
More than two visits	531	22.1
Distance from residence to the hospital (km)		
1–20	1770	73.8
21–100	467	19.5
> 100	162	6.8

### Patients’ satisfaction level with laboratory service

#### Overall satisfaction level

The overall satisfaction level of patients with public hospital laboratory services was 78.6%, the rest customers (21.4%) were dissatisfied with the laboratory services. More than half of the patients were dissatisfied with blood collection processes (many needle stick attempts), poor cleanness of latrine, poor facilities or arrangements to put personnel things during sample collection on the other hand they satisfied with the presence of lab personnel during working hour at reception, courtesy of laboratory personnel, and price.

#### Patients’ satisfaction with access to facilities

According to our finding, nearly 19, 22 and 21% of the respondents could not find the location of the laboratory, cashier office and latrine easily, respectively. They complained that they lost a long time by searching for the locations and feel very disappointed (see Table 3).

#### Patients’ satisfaction with cost of service

The cost of service is crucial to the users of public hospitals, they may have a positive perception or not for the provided service. This study indicated that 83% of the respondents (64% of them said the cost was fair and 19% served freely) were satisfied with the payment of the services, while 17% of the respondents perceived that laboratory test charges were not fair (see Table 3).

#### Patients’ satisfaction with sample collection process

Nearly, 82% of the participants received all the requested laboratory services. Regarding the availability of staff during working hour, 77.3% of the participants were satisfied. The patients’ perception of courtesy during interaction with lab personnel, 67.3% of them were satisfied. Concerning adequacy and availability of sitting arrangement in the waiting area, 45 and 25% of the respondents were satisfied, and dissatisfied respectively. Almost 63% of the respondents were satisfied with the cleanness of the waiting area. Nearly half of the respondents (47.3%) were dissatisfied with the cleanness of the latrine during the survey period (Table 2).

**Table 2:** Participants’ satisfaction level with different laboratory services at selected public hospitals in Ethiopia, November 2017

Satisfaction Characteristics	Number (%)		
	Dissatisfied	Neutral	Satisfied
Cleanness of waiting area	302(12.6)	599(25)	1498 (62.5)
Adequacy of sitting arrangement in the waiting area	599 (24.9)	718 (29.9)	1082 (45.1)
Availability of lab staffs on working hours at the reception area	153 (6.4)	393 (16.3)	1853 (77.3)
Provision of clear orientation during arrival time at sample collection area, why they are coming to the laboratory	636 (26.51)	711 (29.6)	1052 (43.89)
Courtesy/respect of the laboratory personnel	206 (8.6)	578 (24.1)	1615 (67.3)
Provision of clear information where, when & how much the specimen has been collected (e.g. stool, urine, sputum …) (*n* = 1788)	318 (17.8)	569 (31.8)	901 (50.4)
Accessibility of the latrine to collect stool and/or urine specimens (*n* = 1745)	362 (20.74)	0	1383 (79.26)
Cleanliness of latrine	824 (47.3)	425 (24.4)	496 (28.3)
Provision of clear information where and when you receive a laboratory report	499 (20.8)	761 (31.7)	1139 (47.5)

#### Patients’ satisfaction with waiting time

Most of the respondents (88.31%) were not informed or aware of how long each test takes to get the result (turnaround time), while the rest respondents were informed about turnaround time. Out of these informed patients, 29.8% of them did not receive their result within the set turnaround time of each test (Table 2).

#### Patient and laboratory personnel communication/ interaction

Out of the total respondents, nearly 67% of them were satisfied with the courtesy of laboratory personnel, and 26% of them were unsatisfied with the orientation or advisory services provided to them before sample collection. When assessing respondents’ satisfaction with the clarity and adequacy of information they got, where, when and how much specimen (stool, urine, sputum) was collected by themselves (*n* = 1788), half of them were satisfied and 17.8% were dissatisfied. In the same way, from the total participants, 47.5 and 20.8%, of the respondents were satisfied and dissatisfied with clearness of information when, where, and how they received their laboratory results, respectively. On the other hand, nearly 12% of the respondents were unsatisfied due to loss of their laboratory report (Tables 2 and 3).

**Table 3:** Participants’ frequency and percentage distribution of laboratory services at selected public hospitals in Ethiopia, November 2017

Characteristics	Patient n (%)	
	No	Yes
Find the laboratory location easily (*n* = 2399)	458 (19.1)	1941 (80.9)
Find the cashier office easily (*n* = 2399)	518 (21.59)	1881 (78.41)
Is the price fair (*n* = 2399)	409 (17)	1990 (83)
The patient left the laboratory, after the bleeding was stopped and confirmed by the lab personnel (*n* = 1921)	489 (25.46)	1432 (74.54)
Develop bruise (*n* = 1921)	1570 (81.73)	351 (18.27)
Find any access to put your personal things during sample collection (*n* = 1939)	1685 (86.90)	254 (13.10)
Obtain information about torn around time (TAT)	1231 (51.3)	1168 (48.7)
Receive lab result within agreed TAT (*n* = 1168)	348 (29.82)	819 (70.18)
Laboratory result lost	2121 (88.41)	278 (11.59)
Get all the lab services that the physician requests	425 (17.72)	1974 (82.28)

#### Patients’ satisfaction with blood sample collection

**Fig. 1:**
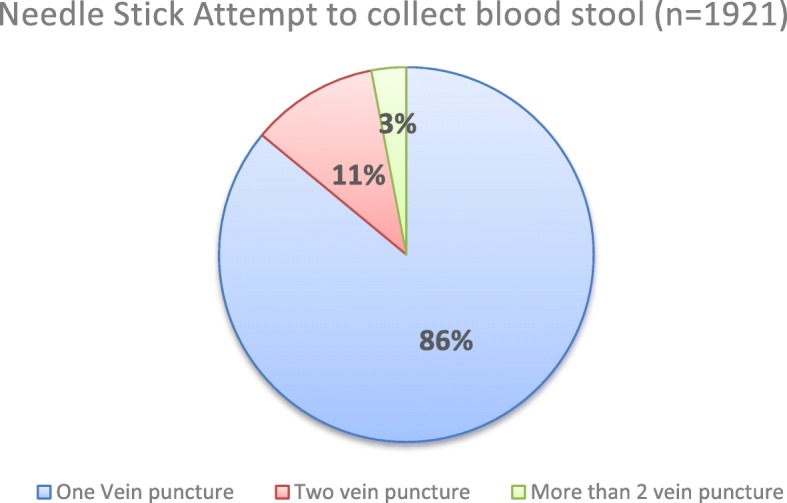
Needlestick attempt during vein puncture from participants to collect a blood sample at selected public hospitals in Ethiopia, November 2017

Nearly, 80% (1921/2399) of the respondents gave a blood sample for different laboratory tests. Out of these respondents, 86.9% of them complained that there was no arrangement in blood drawing room to put their personal belongings in, 25.4% of them left in blood collection area before the bleeding process stopped, 18.27% (351) developed bruise and 14.11% (271) of them punctured more than one times (Fig. 1).

#### Factors that affect satisfaction level of patients

A simple (one outcome and one exposure) logistic regression was used to identify possible explanatory variables and those variables with a *p*-value of less than 0.20, were taken to multiple binary logistic regression model. As a result, educational status (*P* = 0.032), and distance (*P* = 0.000) were significantly associated with patient overall satisfaction level. On the other hand, sex (*P* = 0.149), residence (*P* = 0.25), Occupation (*P* = 0.35) and marital status (*P* = 0.35) were not significantly associated with patient satisfaction level (see Table 4).

**Table 4:** Association of independent variables with a satisfaction level of survey participants at selected public hospitals in Ethiopia, November 2017

Characteristics	Patient Satisfaction		COR^a^(95% CI)	AOR**(95% CI)	*P*-value
	Patient (n)	Dissatisfied (%)			
Sex					
Male	1124	23.13	1.2 (1.0, 1.48)	1.16 (0.95, 1.42)	0.129
Female	1275	19.84	1	1	
Marital Status					
Single	642	20.09	1		0.35
Married	1757	21.85	1.1 (0.89, 1.39)		
Residence					
Urban	1425	22.59	1.2 (0.97, 1.46)	1.13 (0.0.88,1.44)	0.319
Rural	974	19.61	1		
Educational Status					
Illiterate	619	20.35	1	1	0.032
Primary School	675	19.26	0.93 (0.71, 1.22)	0.94 (0.70, 1.25)	
Secondary School	542	20.11	0.98 (0.72,1.31)	1.02 (0.74, 1.40)	
College and Above	563	26.28	1.4 (1.06,1.83)	1.39 (1.02, 1.93)	
Occupation					
Farmer	716	21,65	1.15 (0.88,1.48)		0.468
Gov’t Employed	454	22.91	1.23 (0.92,1.64)		
Private Employed	519	22.35	1.20 (0.90,1.58)		
Others	710	19.43	1		
Number of Visits					
First visit	1209	19.93	1	1	0.067
Two visits	659	21.24	1.08 (0.86,1.37)	1.09 (0.86, 1.38)	
More than two visits	531	24.86	1.33 (1.04,1.69)	1.34 (1.05, 1.72)	
Distance					
Mean 39.86 (3–800)	2399	21.38	1 (1.002,1.004)	1.00 (1.002,1.004)	< 0.001

^a^COR crude odds ratio ^a^AOR adjusted odds ratio

Respondents who served at specialized hospitals were about 5 times (AOR = 4.6; 95% CI = 3.14–6.67) more likely to be dissatisfied than who served at the primary hospital laboratory. Regarding needle stick attempt during blood sample collection, patients who punctured their vein more than 2 attempts were about 3 times (AOR = 2.75; 95% CI = 1.53–4.94) more likely to be dissatisfied than who punctured once (Table 4).

## Discussion

Medical laboratories have a range of customers including patients, physicians, public health agencies, and the community. Measuring patients’ satisfaction level may provide an opportunity to know patients’ concerns about services received views about new services that might be needed. Hence, the current study tried to assess the level of patient satisfaction and outline some possible associated factors with laboratory services at public hospitals.

In this study, the overall patients’ satisfaction level with medical laboratory services was 78.6%. This high satisfaction level could be due to the introduction of social desirability bias by patients. Patients might not be ready to tell their dissatisfaction status freely as the interviews were carried out within the hospitals. This finding was higher than findings of studies conducted in Nekemte Referral Hospital (60.4%), Tikur Anbesa Specialized Hospital (59.7%), St. Paul’s Hospital Millennium Medical College (55.9%), and Pusan National University Hospital, Korea, 70.5% [17, 20–22] and lower as compared with the reports from ART clinics in Addis Ababa (85.5%) and three selected hospitals in Eastern Ethiopia (87.6%) and Iran [23–25]. The discrepancy might be due to differences in the scope of the study, sample size and number of participated health facilities used in previous studies. The current national survey included 60 public hospitals in all regions of Ethiopia and various level of hospitals and 2399 patients. This is the first national study in the country. All previous studies were conducted in one hospital, except for one study that was conducted in three hospitals. Moreover, the current survey included all laboratory services, unlike others that focus only on one service. Higher patients’ satisfaction with ART services may be due to high attention by different partners that implement different interventions of monitoring and follow up procedures.

Accessibility of different hospital facilities like the site of laboratory, latrine, cashier office and others can influence patients’ gratification regarding the hospital service. Hospital facilities are expected to be accessed. However, in the current study, nearly 19, 22 and 21% of the respondents complained that they could not locate the laboratory, cashier office and latrine easily, thereby attenuating their satisfaction. This finding was supported by various studies that showed laboratory patients had low satisfaction level with latrine cleanness and accessibility [23], the convenient location of the laboratory, and the location of the laboratory [20].

Patients would have a negative perception for laboratory service if the requested test was not available, lab staff were not present in the working site, politeness of lab personnel was not good, waiting areas lack sitting facility and were not clean. In the present study, nearly one-fourth up to half of the respondents were dissatisfied with the availability of requested service and lab personnel during their arrival in the laboratory, the courtesy of the laboratory personnel, and the cleanness of the latrine.

Turnaround time (TAT) is the time from when a test is ordered until the result is reported. It is one of the most noticeable signs of laboratory service and is often used as a key performance indicator of laboratory performance. In this study, most of the respondents (88%) were not informed of the turnaround time, and out of informed patients, 29.8% of them did not receive their result within the agreed time. This finding is supported with a study conducted in Addis Ababa, public hospital ART clinics [23], and Hawassa University [26], that showed long waiting hours were associated with dissatisfaction of patients. Therefore, the laboratory, in consultation with the users, should establish turnaround times for each of its examinations that reflect clinical needs and periodically evaluate whether or not it is meeting the established TAT. Monitoring TAT is the ideal choice of activity to illustrate the laboratory’s commitment to providing a high quality service. Improved TAT can be key to greater client satisfaction with the laboratory [27].

Clear and smooth communication is also vital for patient satisfaction. If a patient feels estranged, uninformed about the service and outcomes, it may affect the improvement of their health status. An efficient communication system with the patient of the laboratory is necessary. In the present study, 26% of the respondents were unsatisfied with the explanation or advisory services provided for them before sample collection, nearly 18% of the participants did not get clear and adequate information, where, when and how much specimen (stool, urine, sputum) has been collected by themselves. In the same way, 20.8% of the respondents did not inform clearly when, where, and how they will receive their laboratory results. Our finding is supported by a report from Tanzania that revealed patients feel worried and nervous wondering what clinical examination they are going to undergo, and demand an adequate explanation of the samples they provide and test from laboratory personnel [28]. As a provider of health care services, clinical laboratory technicians have a responsibility to meet their patients’ demands. It is very important for laboratory personnel to provide patients with an explanation in a caring and considerate manner, making it simple and easy-to-understand.

The current study showed that among respondents who gave blood sample, 86.9% of them had a complaint that there was no arrangement in the blood drawing room to put their personal belongings, 25.4% of them left the blood collection area before the bleeding process stopped, 18.27% developed bruise and 14.11% of them punctured more than one times. This finding was supported with a study from Tikur Anbesa Specialized Hospital that showed 81% of laboratory patients said there was no place in the blood drawing room to put personal belongings and 26% of the patients had more than one needle stick attempt during blood collection [20]. Another report from American Opinion Research indicated that blood collection can be a source of significant anxiety for patients even when procedures go well. The report also showed that more than three out of four respondents reported having laboratory personnel experience difficulty drawing their blood [29]. The blood collection procedure is one of the factors that affect the satisfaction level of patients. Therefore, it is critical for medical laboratories to train blood collectors well to make the procedure more comfortable for the patient.

### Limitation

Since patients were interviewed in the hospital setting, they may give responses favoring the care provider resulting in social desirability bias. In addition, no satisfaction study has ever been published from private laboratories in our country, we cannot compare our finding with private laboratories’ service. Our study is limited to our patients: a more powerful design would have been to evaluate other laboratory customers’ satisfaction level. Ultimately it remains to use the results of our evaluations in order to improve the weakest points of our services.

## Conclusion

The overall level of patients’ satisfaction with laboratory service in public hospitals was high. However, a significant number of patients had low satisfaction rate with the accessibility of laboratory and latrine location, adequacy of waiting area, cleanness of latrine, clear and understandable advisory service during specimen collection, long waiting time, inadequate test menu, unfair payment of service, accessibility of any arrangement to put personal things during sample collection, provision of clear information where and when receive a laboratory report, provision of clear information where, when & how much specimen has been collected, cleanness of waiting area, courtesy/respect of the laboratory personnel, availability of lab staffs on working hours at the reception area, blood collection procedure and loss of lab result. Therefore, all responsible bodies in each level should act on the identified gaps and improve the need of laboratory patients’ in each laboratory. In addition, Laboratory personnel should be trained to be courteous and competent as well as the laboratory should conduct regular satisfaction survey to meet the needs of all patients and provide feedback for continuous quality improvement. This national survey of its kind in Ethiopia provided credible evidence to improve the quality of laboratory service and enhancing patient’s satisfaction level and the finding also serves as a baseline data at the national level to evaluate any intervention which is designed to improve the quality of laboratory service.

## Data Availability

All data generated or analyzed during this study are included in this published article.

## Abbreviations

EPHI: Ethiopian Public Health Institute
TAT: Turnaround time
